# miR-365 Ameliorates Dexamethasone-Induced Suppression of Osteogenesis in MC3T3-E1 Cells by Targeting HDAC4

**DOI:** 10.3390/ijms18050977

**Published:** 2017-05-04

**Authors:** Daohua Xu, Yun Gao, Nan Hu, Longhuo Wu, Qian Chen

**Affiliations:** 1 Department of Pharmacology, Guangdong Medical University, Dongguan 523808, China; daohuaxu@gdmu.edu.com; 2 Department of Orthopaedics, Warren Alpert Medical School of Brown University/Rhode Island Hospital, Providence, RI 02903, USA; yun_gao@brown.edu (Y.G.); nan_hu@brown.edu (N.H.); fjwlhwlh@hqu.edu.cn (L.W.); 3 Department of Rheumatology, the First Affiliated Hospital of Xi’an Jiaotong University, Xi’an 710061, China; 4 College of Pharmacy, Gannan Medical University, Ganzhou 341000, China; 5 Bone and Joint Research Center, the First Affiliated Hospital and Frontier Institute of Science and Technology, Xi’an Jiaotong University, Xi’an 710061, China

**Keywords:** miR-365, glucocorticoid, osteoporosis, histone deacetylase 4

## Abstract

Glucocorticoid administration is the leading cause of secondary osteoporosis. In this study, we tested the hypotheses that histone deacetylase 4 (HDAC4) is associated with glucocorticoid-induced bone loss and that HDAC4 dependent bone loss can be ameliorated by miRNA-365. Our previous studies showed that miR-365 mediates mechanical stimulation of chondrocyte proliferation and differentiation by targeting HDAC4. However, it is not clear whether miR-365 has an effect on glucocorticoid-induced osteoporosis. We have shown that, in MC3T3-E1 osteoblasts, dexamethasone (DEX) treatment decreased the expression of miR-365, which is accompanied by the decrease of cell viability in a dose-dependent manner. Transfection of miR-365 ameliorated DEX-induced inhibition of MC3T3-E1 cell viability and alkaline phosphatase activity, and attenuated the suppressive effect of DEX on runt-related transcription factor 2 (Runx2), osteopontin (OPN), and collagen 1a1 (Col1a1) osteogenic gene expression. In addition, miR-365 decreased the expression of HDAC4 mRNA and protein by direct targeting the 3′-untranslated regions (3′-UTR) of HDAC4 mRNA in osteoblasts. MiR-365 increased Runx2 expression and such stimulatory effect could be reversed by HDAC4 over-expression in osteoblasts. Collectively, our findings indicate that miR-365 ameliorates DEX-induced suppression of cell viability and osteogenesis by regulating the expression of HDAC4 in osteoblasts, suggesting miR-365 might be a novel therapeutic agent for treatment of glucocorticoid-induced osteoporosis.

## 1. Introduction

Osteoporosis is a common bone disease characterized by low bone mass and bone structure deterioration, leading to bone fragility and fractures [1]. Glucocorticoid therapy is an important approach for managing inflammatory and autoimmune disorders [2,3]; however, long-term glucocorticoid therapy has several adverse effects including osteoporosis [3,4]. For instance, glucocorticoid administration is the leading cause of secondary osteoporosis [5]. Therefore, it is imperative to develop new drug therapy to counteract glucocorticoid-induced osteoporosis.

MicroRNAs (miRNAs) are small (approximately 22–24 nucleotides) non-coding RNAs that regulate gene expression at the post-transcriptional level by targeting mRNAs via binding to complementary sequences in 3′-UTR. Recent studies showed that miR-24 and miR-23a/b significantly inhibited osteogenic differentiation in bone mesenchymal stem cells (BMSCs) [6,7]. MiR-21 overexpression reversed osteoporosis by targeting RECK [8] and miR-34a prevented osteoporosis by inhibiting osteoclastogenesis via targeting Tgif2 [9]. Thus, microRNAs play important roles in bone development and skeletal disorders. 

Our previous studies showed that miR-365 is a mechanosensitive miRNA and miR-365 stimulates cell proliferation and differentiation by targeting histone deacetylase 4 (HDAC4) in chondrocytes [10,11]. We hypothesize that miR-365 may also enhance osteoblast viability and differentiation by targeting HDAC4 in bone cells, and by doing that, miR-365 ameliorates the glucocorticoid inhibition of osteoblast differentiation. In the present study, we have investigated the effect of miR-365 on dexamethasone (DEX)-induced suppression of osteogenesis in MC3T3-E1 cells. The results showed that miR-365 ameliorated DEX-induced suppression of osteogenesis via a direct interaction between miR-365 and the 3′-UTR of HDAC4 mRNA in osteoblasts, suggesting that miR-365 may be considered a promising therapeutic agent to treat glucocorticoid-induced osteoporosis.

## 2. Results

### 2.1. Dexamethasone (DEX) Inhibited Cell Viability and Decreased the Expression of miR-365 in MC3T3-E1 Cells

**Figure 1:**
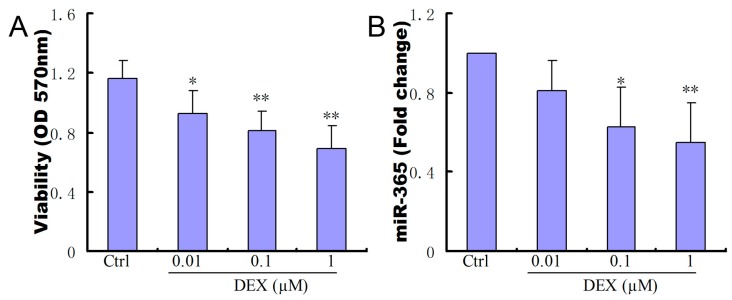
Dexamethasone (DEX) inhibited cell viability and decreased the expression of miR-365 in MC3T3-E1 cells. (**A**) DEX inhibited osteoblast cell viability in a dosage dependent fashion (*n* = 6); (**B**) DEX treatment decreased the expression of miR-365 in MC3T3-E1 cells in a dosage dependent fashion (*n* = 3). * *p* < 0.05, ** *p*< 0.01 compared to control.

We examined the effects of DEX on the viability of MC3T3-E1 cells. The addition of DEX inhibited the viability of MC3T3-E1 cells in a dosage dependent manner (Figure 1A). We also studied the effect of DEX on miR-365 expression. qPCR results showed that DEX treatment significantly reduced miR-365 expression in MC3T3-E1 cells in a dosage dependent manner (Figure 1B).

### 2.2. MiR-365 Over-Expression Ameliorated DEX-Induced Inhibition of Osteoblast Cell Viability and Alkaline Phosphatase (ALP) Activity

**Figure 2:**
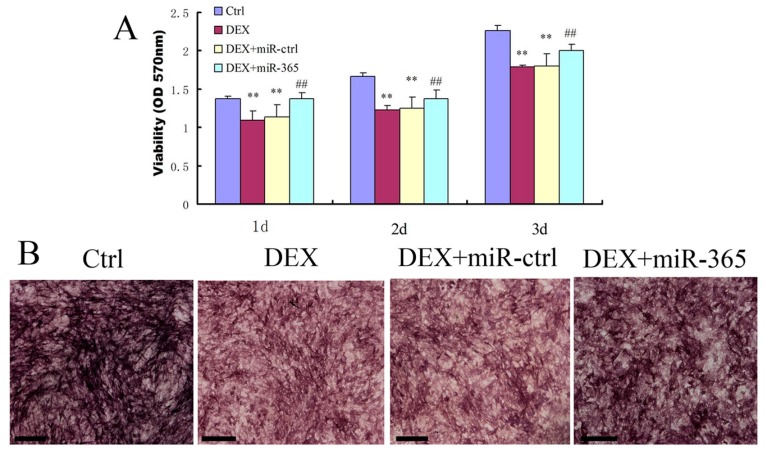
MiR-365 over-expression ameliorated DEX-induced inhibition of osteoblast cell viability and alkaline phosphatase activity. The cells were transfected with miR-365 mimic or miRNA mimic negative control. After 12 h, the cells were treated with 1 µM DEX or vehicle control. (**A**) CCK-8 assay was performed to test the viability on days 1, 2, and 3 (*n* = 6). (**B**) Alkaline phosphatase activity was detected by BCIP/NBT staining on day 7. ** *p* < 0.01 compared to control; ^##^ *p* < 0.01 compared to DEX+miR-ctrl. Scale bar: 200 µm.

To determine whether miR-365 is sufficient to affect cell viability, miR-365 mimic was transfected into MC3T3-E1 cells. While DEX treatment significantly inhibited the viability of MC3T3-E1 cells, miR-365 over-expression significantly prevented cell viability suppression by DEX at one, two, and three days respectively (Figure 2A). In addition, we detected the effect of miR-365 on ALP activity. MC3T3-E1 cells were incubated in osteogenic medium with or without 1 µM DEX after transfection with miR-365 mimic or miRNA mimic negative control. ALP staining was performed by BCIP/NBT solution on day 7. The result showed that miR-365 over-expression ameliorated DEX-induced inhibition of ALP activity (Figure 2B).

### 2.3. MiR-365 Over-Expression Attenuated the Suppressive Effect of DEX on Osteogenic Genes Expression in MC3T3-E1 Cells

**Figure 3:**
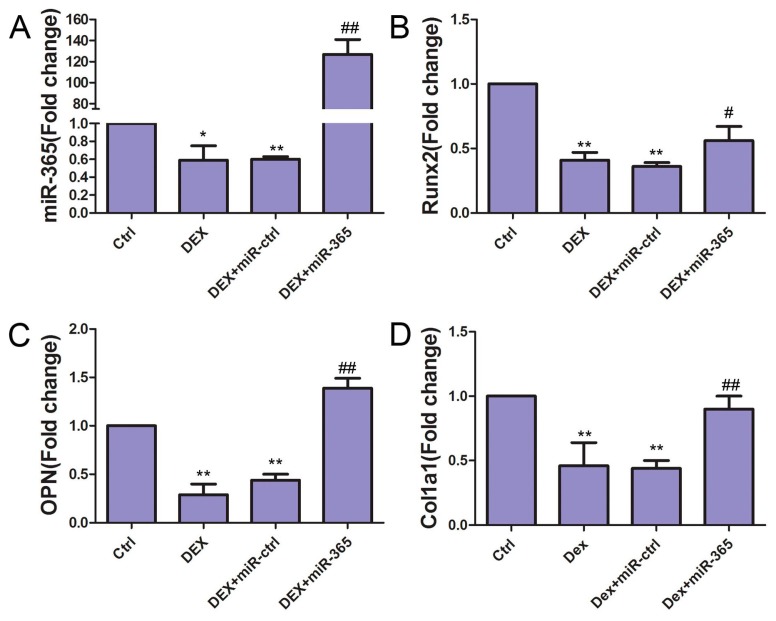
MiR-365 over-expression attenuated the suppressive effect of DEX on osteogenic gene expression in MC3T3-E1 cells. qPCR revealed that the mRNA expressions of miR-365 (**A**), Runx2 (**B**), OPN (**C**), and Col1a1 (**D**) in MC3T3-E1 cells were suppressed by DEX treatment while miR-365 over-expression rescued such suppression. *n* = 3.* *p* < 0.05, ** *p* < 0.01 compared to control; ^#^ *p* < 0.05, ^##^ *p* < 0.01 compared to DEX+miR-ctrl.

To study the effect of miR-365 on DEX-induced suppression of osteogenic differentiation, we detected the osteogenic genes: Runx2, OPN and Col1a1 expressions in MC3T3-E1 cells. MC3T3-E1 cells were cultured to 80% confluence and transfected with miR-365 mimic or miRNA mimic negative control. Then MC3T3-E1 cells were incubated in osteogenic medium with or without 1 µM DEX for three days. Total RNA was extracted for real-time quantitative PCR. qPCR results showed that, while DEX inhibited the mRNA expressions of Runx2, OPN, and Col1a1, miR-365 attenuated the suppressive effect of DEX on all three osteogenic genes (Figure 3).

### 2.4. MiR-365 Over-Expression Inhibited the Upregulation of HDAC4 Induced by DEX

**Figure 4:**
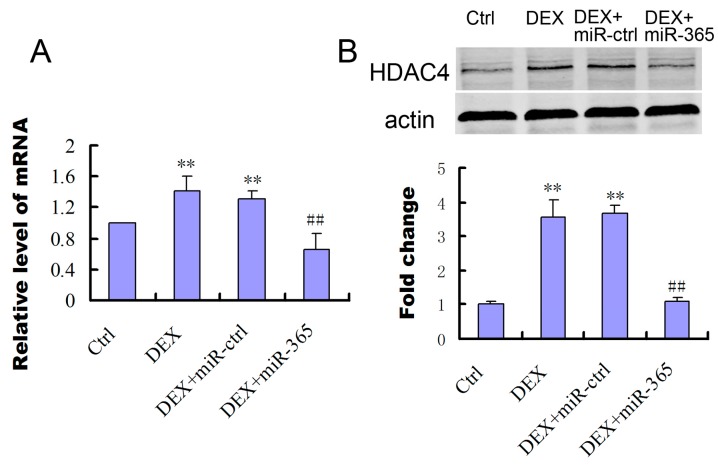
MiR-365 over-expression inhibited DEX stimulation of histone deacetylase 4 (HDAC4) expression. (**A**) mRNA expression of HDAC4 in MC3T3-E1 cells after transfection of miR-365 mimic or negative control (*n* = 3); (**B**) Western blot analyzed the protein expression of HDAC4 in MC3T3-E1 cells after treatment with miR-365 mimic or negative control for 48 h (*n* = 3). ** *p* < 0.01 compared to control, ^##^ *p* < 0.01 compared to DEX+miR-ctrl.

To study the involvement of HDAC4 in Dex treated osteoblasts, we determined that levels of HDAC4 mRNA and proteins (Figure 4). While DEX increased the expression of HDAC4 mRNA and protein in MC3T3-E1, over-expression of miR-365 inhibited the upregulation of HDAC4 induced by DEX (Figure 4).

### 2.5. MiR-365 Directly Targets HDAC4 mRNA in MC3T3-E1 Cells

**Figure 5:**
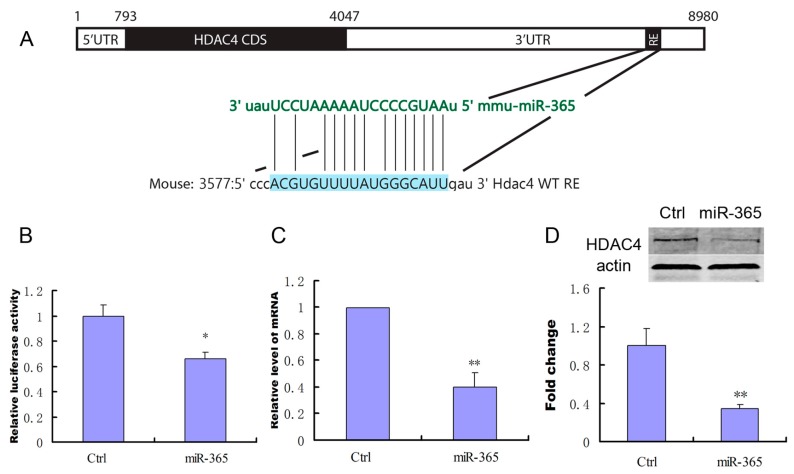
Histone deacetylase 4 (HDAC4) is a direct target of miR-365 in MC3T3-E1 cells. (**A**) Schematic diagram of putative miR-365 seeding-site (Response Element, RE) in 3′-UTR of HDAC4 mRNA; (**B**) pmirGLO-HDAC4 3′-UTR wild-type gene was co-transfected with the negative control or miR-365 mimic into MC3T3-E1 cells respectively. Cells were harvested for quantification of dual luciferase activities at 24 h post transfection (*n* = 3); (**C**) qPCR analysis of the changes of the mRNA expression of HDAC4 in MC3T3-E1 cells after treatment with miR-365 mimic or negative control (*n* = 3); (**D**) Western blot analysis of the changes in HDAC4 protein in MC3T3-E1 cells after treatment with miR-365 mimic or negative control for 72 h (*n* = 3). * *p* < 0.05, ** *p* < 0.01 compared to control.

To investigate whether HDAC4 is a direct target of miR-365 in MC3T3-E1 cells, a wild-type mouse HDAC4 3′-UTR fragment containing miR-365-binding sequence (Figure 5A) was cloned into a luciferase reporter vector, pmirGLO. pmirGLO carrying wild-type HDAC4 3′-UTR constructs was co-transfected with miR-365 mimic or mimic negative control into MC3T3-E1 cells. The relative luciferase activity of the reporter that contained wild-type 3′-UTR was significantly decreased when miR-365 mimic was co-transfected into MC3T3-E1 cells (Figure 5B). Furthermore, transfection of miR-365 decreased the expression of the mRNA and protein of HDAC4 (Figure 5C,D). These results indicate that miR-365 can directly suppress HDAC4 expression by targeting 3′-UTR in MC3T3-E1 cells.

### 2.6. MiR-365 Increased Runx2 Expression and HDAC4 Over-Expression Inhibited this Effect

**Figure 6:**
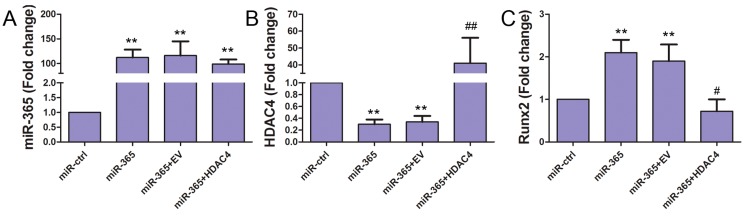
MiR-365 increased Runx2 expression and Histone deacetylase 4 (HDAC4) over-expression inhibited this effect of miR-365. The MC3T3-E1 cells were co-transfected with miRNA mimic control (miR-ctrl); miR-365 mimic (miR-365); miR-365 and a cDNA plasmid empty vector (EV) (miR-365+EV); miR-365 and a vector containing HDAC4 cDNA (miR-365+HDAC4). The day before transfection, MC3T3-E1 cells were cultured to 80% confluence. Transfection was performed using Lipofectamine 3000 (Invitrogen) according to the manufacturer’s instructions. 48 h after transfection, RNA levels of miR-365 (**A**), HDAC4 (**B**), and Runx2 (**C**) was quantified by qPCR. *n* = 3. ** *p* < 0.01 compared to miR-ctrl; ^#^ *p* < 0.05, ^##^ *p* < 0.01 compared to miR-365+EV.

Runx2 plays an important role in osteoblast differentiation [12]. To determine whether miR-365 regulates Runx2 in MC3T3-E1 cells, we quantified the Runx2 mRNA level. MiR-365 transfection significantly increased the expression of Runx2 mRNA. Co-transfection of HDAC4 cDNA significantly inhibited the increase of Runx2 promoted by miR-365 (Figure 6). These data suggest that miR-365 can regulate the expression of Runx2 via inhibition of the HDAC4 pathway.

## 3. Discussion

Glucocorticoids are widely used in the treatment of inflammatory and autoimmune diseases. However, long-term glucocorticoid therapy can lead to reduction in bone mass [5,13]. Glucocorticoid-induced osteoporosis is the third most common type of osteoporosis, preceded by postmenopausal and senile osteoporosis [14]. DEX is a commonly used glucocorticoid and studies have shown that DEX inhibited osteogenic differentiation and bone formation [15,16]. In this study, we showed that DEX can decrease the viability and ALP activity of MC3T3-E1 and miR-365 can significantly reverse this suppressive effect of DEX on MC3T3-E1. Furthermore, we found that DEX inhibited the expression of Runx2, OPN, and Col1a1 in MC3T3-E1 and miR-365 significantly ameliorated the suppressive effect of DEX on osteogenetic genes. Thus, miR-365 may serve as a new therapeutic agent for counteracting the inhibition of glucocorticoid-induced osteoblastic differentiation.

HDACs are a family of enzymes that catalyze the removal of acetyl groups from lysine residues in histones and non-histone proteins and they play a key role in the transcriptional regulation of gene expressions [17,18]. Studies have shown that HDAC4 has a vital role in skeleton formation [19,20]. Mice with a global deletion of HDAC4 display ectopic ossification of endochondral cartilage [21]. HDAC4 participates in parathyroid hormone (PTH)-induced bone metabolism [22] and histone deacetylase inhibitors promote osteoblast differentiation [23,24]. Our data showed that DEX increased the expression of HDAC4 and miR-365 inhibited the promotion of HDAC4 led by DEX. Furthermore, we found that miR-365 directly targets conserved seeding sites within the 3′-UTR of HDAC4. Therefore, HDAC4 is the target, by which miR-365 regulates the glucocorticoid suppression of osteoblast differentiation. In addition, Ko et al. have shown that glucocorticoid promoted the expression of HDAC4 and miR-29a regulated excess glucocorticoid suppression of osteoblast differentiation by targeting HDAC4 [25]. Thus, HDAC4 may be a common target regulated by multiple miRNAs during glucocorticoid suppression of osteoblast differentiation.

In this study, we showed that miR-365 stimulates Runx2, an essential transcription regulator that plays a crucial role in osteoblast differentiation [12,26]. Furthermore, we showed that miR-365 stimulation of Runx2 is mediated by its knockdown of HDAC4 in osteoblasts. Studies have revealed that both intramembranous and endochondral ossification are completely blocked in Runx2 null mice and overexpression of Runx2 can enhance osteoblastic differentiation [26,27]. Furthermore, studies have shown that HDAC4 regulates Runx2 activity [28]. Cao et al. have shown that HDAC4 inhibited Runx2 promoter activity in a human chondrocyte cell line [29]. Smith et al. showed that miR-365 is involved in osteoblastic differentiation in B6 and C3H cells [30]. We have previously shown that miR-365 promotes chondrocyte proliferation and differentiation by inhibiting HDAC4 in chondrocytes [11]. Thus, a miR-365/HDAC4/Runx2 axis may be involved in regulating both chondrocyte and osteoblast differentiation. 

**Figure 7:**
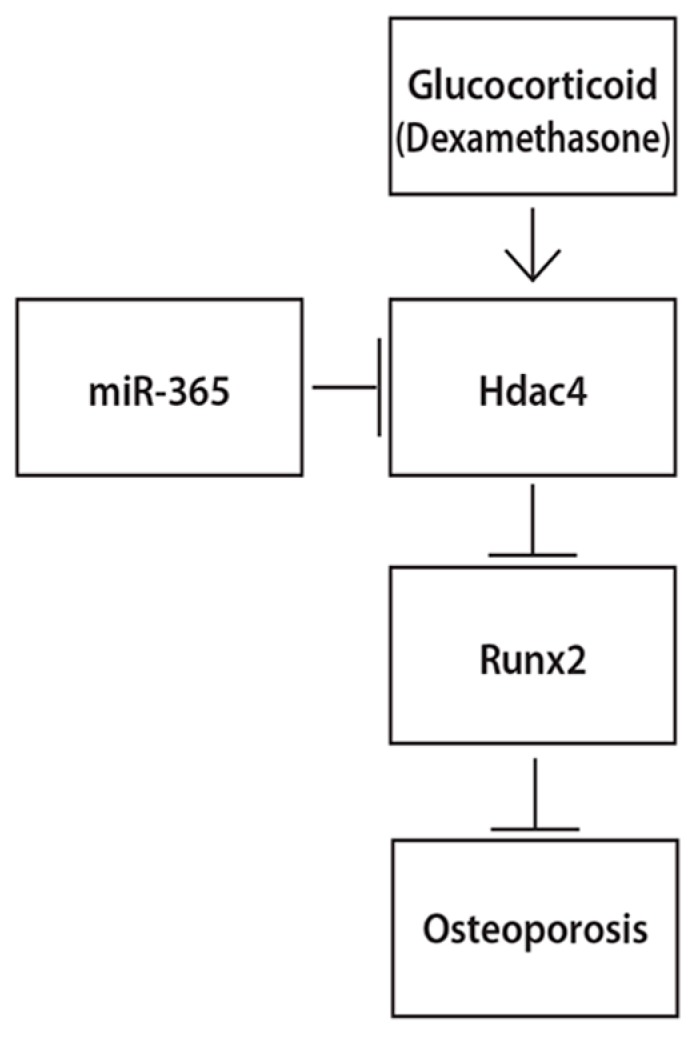
Schematic diagram showing the possible mechanisms by whichmiR-365 ameliorates DEX-induced osteoporosis. → means increase, ┫ means inhibition.

Our data suggest that such a regulatory axis may be used for the treatment of glucocorticoid induced bone loss (Figure 7). In this present study, we found that DEX inhibited the expression of Runx2 while miR-365 attenuated such inhibition by DEX. MiR-365 upregulated the expression of Runx2 through direct targeting HDAC4 in osteoblasts. Therefore, Runx2 is involved in the effect of miR-365 for counteracting the suppression of osteoblast differentiation induced by glucocorticoid. In conclusion, our results collectively indicate that miR-365 ameliorates DEX-induced suppression of osteogenesis by directly regulating HDAC4. MiR-365 may be a potent therapeutic agent for the prevention and treatment of glucocorticoid-induced osteoporosis.

## 4. Materials and Methods

### 4.1. Cell Culture

MC3T3-E1 cells were purchased from ATCC and grown in Modified Eagle’s Medium of α (α-MEM) (Gibco, Waltham, MA, USA) supplemented with 10% fetal bovine serum (FBS) (Gibco, Waltham, MA, USA), 100 U/mL penicillin and 100 µg/mL streptomycin (Gibco, Waltham, MA, USA). For the induction of osteoblastic differentiation, MC3T3-E1 cells were incubated in osteogenic medium (α-MEM, 10% fetal bovine serum, 10 mM β-glycerophosphate, and 50 µg/mL ascorbic acid) and treated with 1 µM DEX (Sigma, St. Louis, MO, USA) or vehicle control. The medium was changed every three days.

### 4.2. MiRNA Transfection

MC3T3-E1 cells were cultured to 80% confluence and transfected with miR-365 mimic (Dharmacon, Lafayette, CO, USA) or miRNA mimic negative control (Dharmacon, Lafayette, CO, USA) by Lipofectamine 3000 (Invitrogen, Waltham, MA, USA) according to the manufacturer's instructions. MiR-365 mimic and miRNA mimic negative control were used at a final concentration of 50 nM.

### 4.3. Cell Viability Assay

Cell viability was measured using the Cell Counting Kit-8 (CCK-8, Sigma, St. Louis, MO, USA). Briefly, samples were sub-cultured in a 96-well plate and thecells were transfected with miR-365 mimic or miRNA mimic negative control. 12 h later, the cells were treated with 1 µM DEX or vehicle control for one, two, or three days. The cell viability was assessed by the CCK-8. The absorbance at 450 nm was measured by a microplate reader (SpectraMAX Me^2^, Molecular Device, Sunnyvale, CA, USA).

### 4.4. ALP Staining

ALP staining was performed by using BCIP/NBT solution (Sigma, St. Louis, MO, USA). Briefly, the treated cells were washed with phosphate buffer saline (PBS) twice and fixed with 70% ethanol for 10 min. The cells were equilibrated by ALP buffer (0.15 M NaCl, 0.15 M Tris-HCl, 1 mM MgCl_2_, PH 9.5) twice, incubated with NBT-BCIP solution at 37 °C in dark for 30 min. Then the reaction was stopped by distilled water and the plate was dried before taking photos.

### 4.5. RNA Extraction and Real-Time Quantitative PCR

Total RNA was extracted from cultured cells with miRNeasy RNA Mini Kit (Qiagen, Germantown, MD, USA) according to the manufacturer’s instructions. For miRNA detection, RNA was reverse transcribed using miScriptIIRT Kit (Qiagen, Germantown, MD, USA). The ubiquitously expressed miRNA, snRNA U6, was used as an endogenous control.For mRNA detection, RNA was reversely transcribed using iScript cDNA Synthesis Kit (Bio-Rad, Hercules, CA, USA). The miRNA and mRNA levels were quantified by real-time quantitative PCR (qPCR) with the SYBR Green PCR Master mix (Qiagen, Germantown, MD, USA). Quantitative real-time PCR was performed by using a Bio-Rad CFX96 real-time PCR detection system (Bio-Rad, Hercules, CA, USA).Amplification conditions were as follows: 95 °C for 10 min, 40 cycles of 95 °C for 10 s, 55 °C for 30 s, and 72 °C for 30 s. Sense and antisense primers were as follows: Runx2 forward: 5′-agatgacatccccatcccatc-3′, reverse: 5′-gtgagggatgaaatgcttgg-3′; OPN forward: 5′-cactccaatcgtccctaca-3′, reverse: 5′-gctgccctttccgttgtt-3′; Col1a1 forward: 5′-ttaggggccactgccctcct-3′, reverse: 5′-gcactcgccctcccggcttt-3′; HDAC4 forward: 5′-gtgaagcaggagcccatt-3′, reverse: 5′-ggagggcttgctgtctga-3′; 18S forward: 5′-cggctaccacatccaaggaa-3′, reverse: 5′-gctggaattaccgcggct-3′; 18S ribosomal RNA was used as an internal control gene to normalize the mRNA levels. These primers were synthesized by Intergrated DNA Technologies. The primers for miR-365 were purchased from Qiagen. Fold changes of mRNA and miRNAs were calculated by the 2^–ΔΔ*C*t^ method and normalized to 18S or U6 snRNA, respectively.

### 4.6. Luciferase Assays

MC3T3-E1 cells were cultured at 1 × 10^5^ cells/well in 12-well plates. The cells were co-transfected with miR-365 mimic (50 nM) or miRNA mimic negative control (50 nM) and 0.2 µg of pmirGLO-HDAC4 3′-UTR. Transfection was performed using Lipofectamine 3000 (Invitrogen, Waltham, MA, USA). After 48 h, cells were collected and luciferase activity was determined using the Dual-Luciferase reporter assay system (Promega, Madison, WI, USA) with the dual luciferase assay reporter-ready luminometer (Promega, Madison, WI, USA). The assays were performed in triplicate.

### 4.7. Western Blot

All pre-treated samples were washed with PBS and lysed in lysis buffer (M-PER, Life Technologies, Waltham, MA, USA) plus protease inhibitor phenylmethylsulfonyl fluoride (Thermo Fisher Scientific, Waltham, MA, USA) for 30 min on ice. The lysates were centrifuged at 12,000× *g* for 15 min at 4 °C. The supernatants were collected and the protein concentrations were determined using BCA assay (Thermo Fisher Scientific, Waltham, MA, USA). After being heated for 5 min at 95 °C, equal proteins(30 µg) for each sample were separated by 10% SDS-polyacrylamide gel and then transferred to polyvinylidene difluoride (PVDF) membrane (Whatman, Lafayette, CO, USA) for 70 min at 100 V. The membrane was blocked with 5% bovine serum albumin (BSA) in Tris-buffered saline-Tween 20 (0.1%) (TBS-T) for 1 h and incubated with anti-HDAC4 or anti-actin antibodies (Abcam, Cambridge, MA, USA) at 4 °C overnight. On the next day the membrane was incubated with anti-rabbit-Alexa Fluor 680 (Molecular Probes, Eugene, OR, USA) for 1 h at room temperature. The blots were scanned using an Odyssey fluorescence scanner (LI-COR Biosciences, Lincoln, NE, USA). The band intensity was quantified using the Odyssey software.

### 4.8. Statistical Analysis

All data were presented as mean ± SD and statistical analysis was performed using one-way analysis of variance (one-way ANOVA) among multiple groups and student’s *t*-test between two groups. A value of *p* < 0.05 was considered statistically significant.

## 5. Conclusions

MiR-365 ameliorates DEX-induced suppression of cell viability and osteogenesis by regulating the expression of HDAC4 in osteoblasts. These findings suggest that miR-365 might be a novel therapeutic agent for treatment of glucocorticoid-induced osteoporosis.
